# Eczema Herpeticum: Clinical Insights and Pathogenesis Hypotheses on Basolateral Adhesion Proteins

**DOI:** 10.7759/cureus.66932

**Published:** 2024-08-15

**Authors:** Jesús Iván Martínez-Ortega, Samantha Franco González

**Affiliations:** 1 Dermatology, Dermatological Institute of Jalisco, Zapopan, MEX; 2 Internal Medicine, XXI Century National Medical Center, Ciudad de México, MEX

**Keywords:** immunological pathogenesis, herpes simplex virus (hsv), eczema herpeticum, basolateral adhesion proteins, skin barrier, immune dysfunction, structural proteins, atopic dermatitis, systemic infection, kaposi varicelliform eruption

## Abstract

Eczema herpeticum (EH) is a severe viral complication caused by the herpes simplex virus (HSV) that occurs in individuals with compromised skin barriers, such as those with atopic dermatitis (AD). EH is characterized by the rapid spread of HSV across skin lesions, potentially leading to systemic involvement. Although commonly observed in the context of AD, EH can also arise in various dermatological conditions, necessitating prompt recognition and management by healthcare providers. This case report details the diagnosis and treatment of EH in a five-year-old girl with a history of AD who presents with fever and painful skin lesions. Despite the absence of confirmatory tests initially, a positive IgM anti-HSV-1 serology, combined with clinical presentation, supported the diagnosis of EH. The patient received intravenous aciclovir, resulting in significant improvement within 48 hours. This case highlights the importance of early diagnosis and treatment, particularly when confirmatory tests are not available.

The report discusses the clinical presentation of EH, which includes vesicular lesions, fever, and rapid progression. The differential diagnosis includes chickenpox, impetigo, eczema vaccinatum, and contact dermatitis. Understanding the epidemiology and pathogenesis of EH, especially in relation to AD, is crucial for effective management. The case also introduces a novel hypothesis linking structural protein alterations to immune dysfunction in EH, suggesting a need for further research. Acyclovir remains the gold standard for treating EH, and timely intervention is essential. This case underscores the necessity of a diagnostic algorithm in the absence of guidelines and highlights the role of IgM serology and clinical judgment in managing suspected EH cases.

## Introduction

Eczema herpeticum (EH) is a severe viral complication of the herpes simplex virus (HSV) that occurs in individuals with compromised skin barriers, such as those with atopic dermatitis (AD). EH is a life-threatening condition characterized by the rapid spread of HSV across skin lesions, potentially leading to systemic involvement. While EH is commonly observed in the context of AD, it can also arise in other dermatological conditions, making it crucial for healthcare providers to recognize and manage it promptly [1-3].

AD, the most prevalent inflammatory skin disease globally, affects up to 25% of children and 10% of adults [1]. Patients with AD are particularly susceptible to viral infections due to their impaired skin barrier and immune dysregulation. EH, caused by HSV, presents as clusters of umbilicated vesicles, fever, and rapid disease progression [2]. Although EH specifically denotes extensive HSV infections in AD, it can also occur in other skin conditions, making differential diagnosis important.

Understanding the epidemiology of EH is essential for effective management. The incidence of EH in AD patients is estimated at around 3%, but its prevalence in other skin conditions is less well-defined [2,3]. 

To differentiate EH from other conditions such as chickenpox, impetigo, eczema vaccinatum, and contact dermatitis, clinicians should carefully consider specific clinical features and patient history. Management of EH requires early identification and treatment with antiviral therapy, primarily aciclovir, to prevent severe complications [2]. Timely and accurate diagnosis by healthcare providers is essential. This case report details the diagnosis and treatment process for EH in the absence of confirmatory tests and presents a hypothesis regarding its pathogenesis.

## Case presentation

A five-year-old girl with a history of AD was admitted to the clinic with fever and painful skin lesions persisting for three days. Diagnosed with AD at age two, she had received all scheduled vaccinations. On examination, her temperature was 38.6°C, her heart rate was 124 beats/min, and her respiratory rate was 24 breaths/min. No palpable lymphadenopathy was noted.

Physical examination revealed multiple diffuse grouped papulovesicles, some eroded and crusted, with corkscrew ulcers on her face, upper limbs, and anterior trunk, accompanied by tenderness. There were no recent exacerbations of AD or additional symptoms.

Initial diagnostic tests

**Figure 1:**
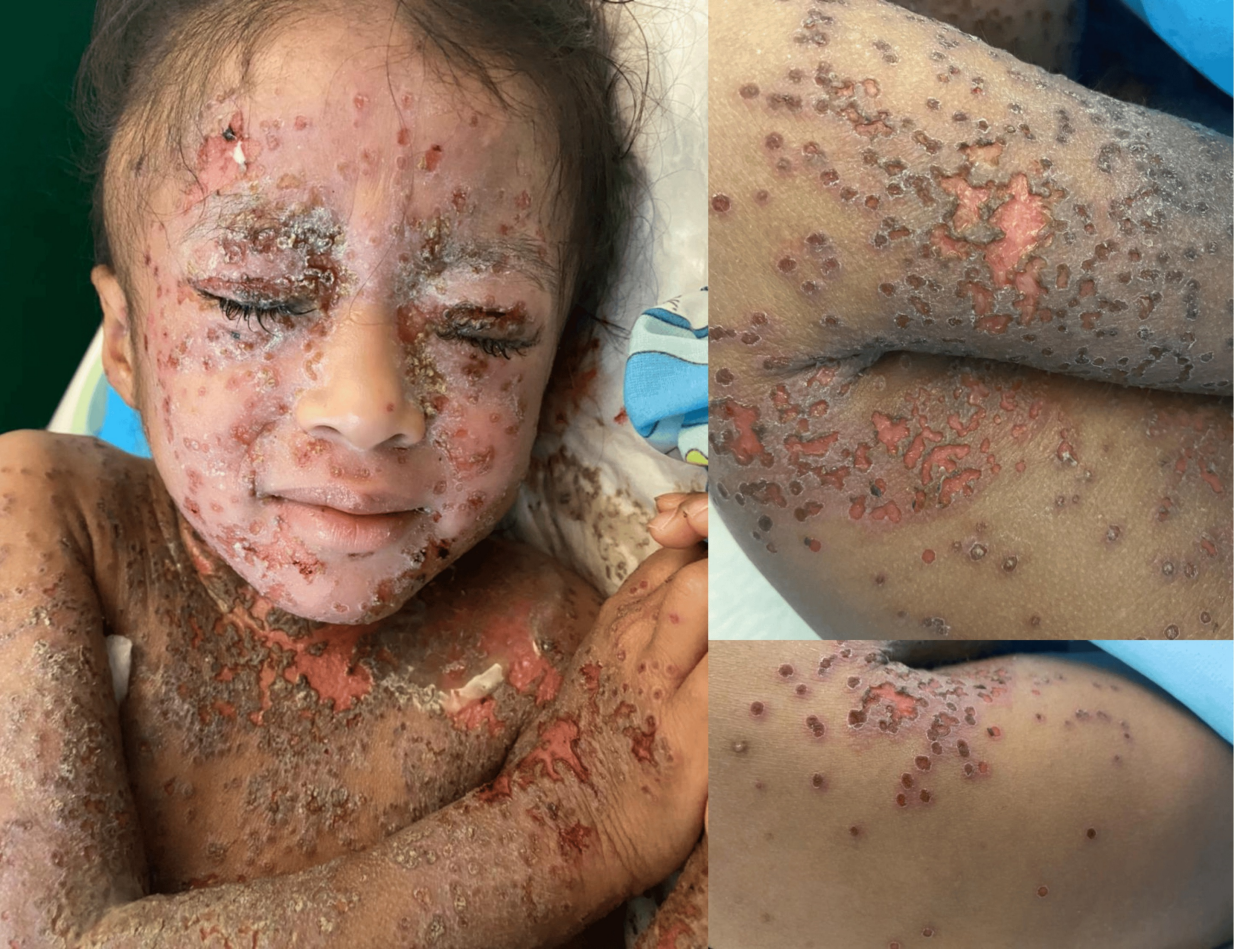
Clinical presentation.

Complete blood count (CBC): Leukocytosis with a count of 13,000x10^3^ (normal range: 4000-10,000×10^3^), basic metabolic panel (BMP): normal, liver function tests (LFTs): normal, renal function tests: normal, serology for IgM and IgG: Epstein-Barr virus, parvovirus, human herpesvirus type 6, coxsackievirus, and cytomegalovirus: negative, serology for IgM anti-HSV-1: positive, serology for IgG anti-HSV-1: negative, polymerase chain reaction (PCR) for HSV: not performed initially, Tzanck test: not performed initially (Figure 1).

Given the clinical presentation and initial diagnostic tests, including the positive IgM anti-HSV-1 serology, HSV infection was suspected. Although PCR and Tzanck tests were not performed initially, they would be recommended for confirmation of EH. The patient was admitted to the emergency department for monitoring and management.

Treatment with intravenous aciclovir (10 mg/kg three times daily for seven days) was initiated. Supportive care included oral fluids for hydration, appropriate analgesics for pain management, emollients, and mupirocin applied to the affected areas. The pediatric team was consulted for comprehensive care, including fever management and hydration.

Late laboratory tests were done on days two and three. The CBC was within the normal range. BMP, LFTs, and renal function tests remained normal. The patient demonstrated significant improvement within 48 hours of treatment initiation and was discharged without complications. Follow-up care was arranged to monitor recovery and address any potential long-term effects.

## Discussion

EH was initially termed 'Kaposi’s varicelliform eruption' (KVH) by Moritz Kaposi in 1887, originally believed to stem from a fungal infection. Although often used interchangeably, the terms KVH and EH are not synonymous [3,4]. Presently, KVH refers to all viral complications associated with HSV in various dermatoses, such as AD, psoriasis, rosacea, pemphigus, and more rarely, conditions like Grover's disease, Hailey-Hailey disease, Darier's disease, epidermolysis bullosa simplex, pityriasis rubra pilaris, and others [3]. EH specifically denotes extensive HSV infections in AD [2], though some argue it can extend to other dermatoses and is not exclusively caused by HSV [3]. In most cases, EH is caused by the reactivation of HSV types 1 and 2. However, it can also rarely be caused by other viruses such as Coxsackie A16, vaccinia, and varicella-zoster [5].

When dome-shaped papulovesicles with crusts and erosions are accompanied by pain and fever, EH should be considered. EH typically presents with clusters of umbilicated blisters, fever, and rapid progression [3,5].

To differentiate EH from other conditions, such as chickenpox, impetigo, eczema vaccinatum, and contact dermatitis in the context of atopic dermatitis, consider the following: Chickenpox features generalized, itchy vesicles appearing in crops; impetigo is characterized by honey-colored crusts; eczema vaccinatum involves severe, widespread vesicular lesions following smallpox vaccination, and contact dermatitis presents with erythema and itching at contact sites. While diagnosis is primarily clinical, supported by patient history and examination, specific tests may be employed in cases of uncertainty when available: PCR or culture for HSV in EH, PCR for varicella, bacterial culture for impetigo, clinical history for eczema vaccinatum, and patch testing for contact dermatitis [3-5].

The pathogenesis of AD involves a dysfunction in the epidermal water barrier located just above the stratum granulosum [1]. This barrier consists of both protein and lipid components. Essential proteins like loricrin and filaggrin are initially synthesized within intracellular keratohyalin granules. As cornification proceeds, these proteins are secreted into the extracellular space, aiding in barrier formation. Additionally, other proteins positioned between the keratinocytes of the stratum granulosum contribute to barrier integrity. To simplify, we will collectively refer to the protein component of the barrier and the proteins involved in cell-to-cell adhesion as structural proteins [6].

Genetic mutations in structural proteins of the epidermal barrier lead to structural protein deficits or malfunctions in filaggrin or claudin, for instance, which leads to an impaired barrier, transepidermal water loss, skin-surface pH alterations, unbalanced skin microbiota, and immune system dysregulation [1,2].

Tight and adherent junction protein mutations such as Claudine or E-cadherin might lead to a “loose” cell-to-cell adhesion, which facilitates virus entry and spread through the epidermis. Interestingly, one of the adherent junction proteins is a receptor for HSV, namely, nectin-1. The disruption in cell-to-cell adhesion would increase the availability of nectin-1 and allow the virus to enter the cell [2,7].

**Figure 2:**
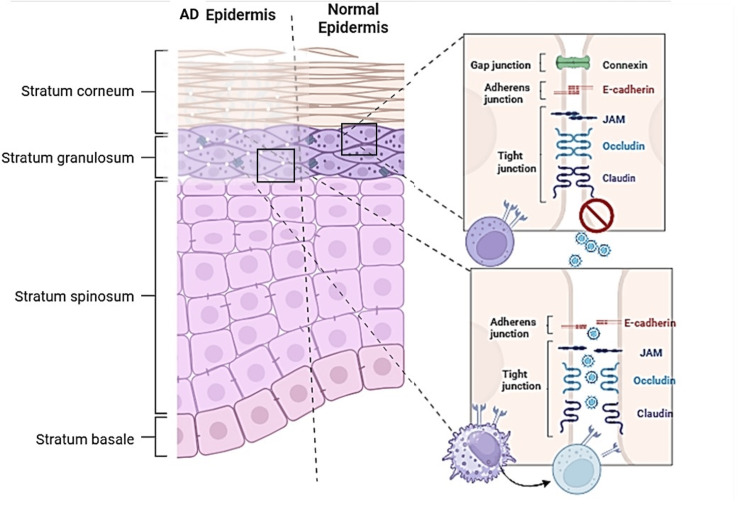
Visual representation of pathogenesis hypothesis. The figure illustrates the epidermis of atopic dermatitis (AD), showing an abnormality characterized by "loose" intercellular spaces due to mutations causing loss of function or deficiency in adhesion proteins. This exposes the adhesion proteins, which also act as both viral receptors and NK cell receptors. Initially, NK cells may become activated, but chronic exposure leads to an exhaustion phenotype characterized by low cytotoxic/antiviral activity and increased proinflammatory response. The upper and right sides of the image depict a healthy epidermis, contrasting with the abnormality seen in AD. Image credits: Jesus Martinez, MD. Created with biorender.com. NK: natural killer.

**Figure 3:**
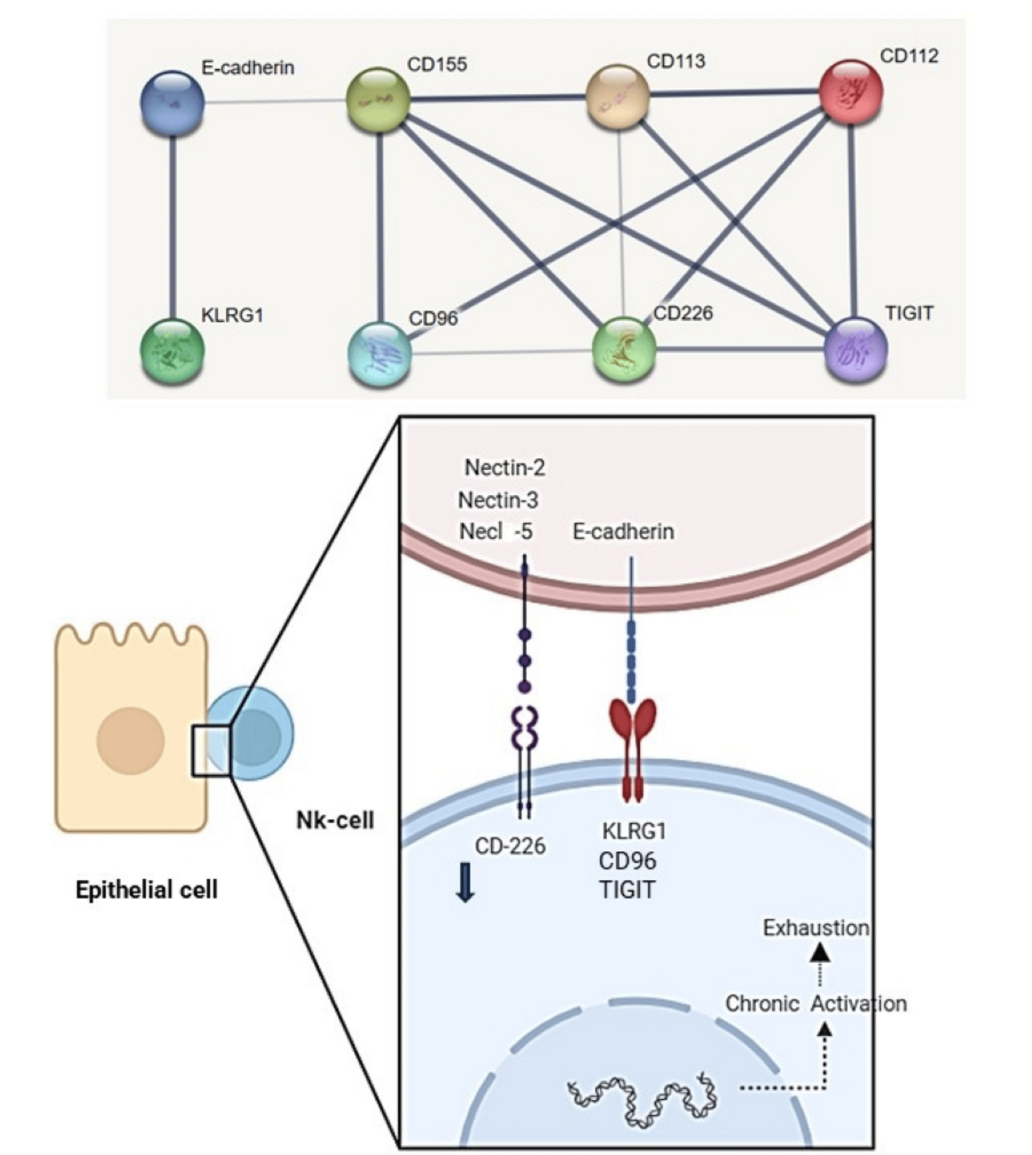
Combined figure of an interactome and a schematic visualization of NK cell receptors and their respective basolateral keratinocyte ligands. The figure showcases an interactome generated using STRING, illustrating the intricate interaction between NK cell receptors and ligands, which are adhesion proteins located on the basolateral epithelial membrane. This depiction reveals the complexity of the relationship, with the potential for multiple receptors to bind to a single ligand, and the presence of both inhibitory and activating receptors. The accompanying image below, created with Biorender.com, features three key members of the nectin and nectin-like family (nectin2 or CD112, nectin3 or CD113, and necl-5 or CD155), which share an activator receptor CD226 and the E-cadherin-binding KRG1 receptor representing the inhibitors receptors. Additionally, there are other inhibitor receptors below that bind basolateral ligands. The blue arrow adjacent to CD226 indicates the downregulation of the activating receptor following persistent stimulation. Furthermore, there are other receptor-ligand interactions not depicted, including JAML-CAR, CRTAM-necl-2, and PVRIG-nectin2. Image credits: Jesus Martinez, MD. JAML: Junctional Adhesion Molecule-Like Protein, CAR: Coxsackie and adenovirus receptor, NK: natural killer.

Interestingly, AD has been related to an accumulation of natural killer (NK) cells with low activating receptors, which showed decreased cytolytic activity but increased TNF-α production and is correlated to further allergen sensitization and diminished skin barrier function [8]. Intriguingly, not only viruses use basolateral adherent proteins as receptors, but NK cells use them as well, for instance, Necl-5 and E-cadherin proteins [9]. Thus, we propose that in AD, the structural alterations that increase the availability of virus receptors may also lead to exhaustion through hyperactivation of NK cell receptors, potentially resulting in reduced antiviral cytolytic activity and compromised immune responses to HSV (Figures 2, 3). This hypothesis warrants further investigation through basic science research.

Despite the limitations of IgM serology, such as its persistence for 48 to 89 days and potential reappearance in subsequent HSV episodes, a positive IgM result is relatively rare in the early phase of infection (33% within seven days of lesion appearance) but can be a crucial diagnostic tool when positivity is present [10]. In this case, the combination of positive IgM serology, clinical presentation, and negative IgG supported the diagnosis of EH. The patient’s rapid improvement with antiviral therapy further corroborated the diagnosis, underscoring the utility of IgM serology in conjunction with clinical assessment in early HSV infection.

Before acyclovir was developed in 1978, up to 50% of EH cases were fatal due to ineffective treatments. Acyclovir, now the gold standard for EH, is administered intravenously at 5-10 mg/kg for seven days and has proven highly effective [3].

Luca et al. identified key predictors for hospitalization in their study: male sex (OR = 3.09; 95% CI, 1.20-7.95, P = 0.017), fever (OR = 5.75; 95% CI, 2.17-15.26, P < 0.001), systemic symptoms (OR = 2.84; 95% CI, 1.06-7.62, P = 0.035), and age under one year (OR = 7.17; 95% CI, 2.17-23.72, P = 0.001) [11]. In this case, the presence of systemic symptoms and fever were consistent with these predictors, supporting their relevance for hospitalization.

The primary limitation of this article is the absence of confirmatory tests such as PCR and the Tzanck test. However, as noted in the table, the sensitivity and specificity of these tests can decrease after three days from lesion onset and in cases with crusted lesions, where viral particles may be significantly reduced [12]. In the absence of established guidelines for eczema herpeticum (EH) and given the gaps in the literature, particularly for chronic or specific lesion types, reliance on clinical features for immediate treatment remains a practical approach when confirmatory tests are unavailable or not feasible in certain contexts (Table 1).

**Table 1:** Positive rates of Tzanck test and PCR. This table, based on information from reference [10], illustrates how the positive rates of the Tzanck test and PCR are affected by the type and duration of lesions. PCR: polymerase chain reaction.

Category	Sub-category	n		Tzanck positive rate	PCR positive rate
Lesion type	Blister		38	81.6%	100%
	Pustule		7	85.7%	100%
	Eroded ulcer		5	40%	60%
	Crust		48	62.5%	43.7%
Days	1–3 days		40	78.7%	80.8%
	4–6 days		47	64%	80.6%
	7–30 days		11	27%	80.7%

## Conclusions

The clinical presentation of EH, characterized by vesicular lesions, fever, and rapid progression, remains essential for diagnosis. Additionally, the presence of fever and systemic symptoms predicts the need for hospitalization. Although IgM serology is not always positive (with about 33% positivity in early infections), a positive result, when correlated with clinical features and a negative IgG, can be an additional supportive tool. Therefore, timely diagnosis and treatment should not be delayed, and clinical judgment remains paramount in managing suspected EH cases.

In the absence of current guidelines and with a limited understanding of EH pathogenesis, this case underscores the necessity of a diagnostic algorithm, particularly when all diagnostic tools are unavailable or prompt action is required. The proposed hypothesis linking structural protein alterations to immune dysfunction offers a novel perspective for future research in the context of EH and AD. Acyclovir, being inexpensive, accessible, and highly effective, remains a crucial component of treatment, especially when a high index of suspicion is present and rapid intervention is necessary.
